# Editorial: Dialogue with robots: constructive approaches for understanding communication

**DOI:** 10.3389/frobt.2025.1725162

**Published:** 2025-11-14

**Authors:** Casey Kennington, Koji Inoue, Randy Gomez, Peter Ford-Dominey

**Affiliations:** 1 Department of Computer Science, Boise State University, Boise, ID, United States; 2 Kyoto University, Kyoto, Japan; 3 Honda Research Institute, Wako-shi, Japan; 4 Centre National de la Recherche Scientifique (CNRS), Paris, France

**Keywords:** dialogue, robots, human-robot interaction, multimodal, speech

## Introduction

This Research Topic brings together contributions focusing on dialogue with robots, with a broader goal of understanding where robots fit into our everyday lives through practical uses as well as how different populations perceive social robots. This line of human-robot interaction (HRI) research moves us closer to aligning human needs with the capabilities of robots. The implications of being able to naturally communicate with robots will affect domains such as healthcare and education, and also enable humans and robots to work together in industrial settings without the need for tedious technical training.

## Frameworks

In Groß et al., the authors present *RISE*, or *Robotics Integration and Scenario-Management Extensible-Architecture,* an open-source framework for reproducible HRI research. RISE addresses challenges in HRI research by offering an accessible and configurable system built on three core structures: (1) communication robot acts, (2) interaction rules, and (3) working memory. RISE offers HRI experiment designers an easy-to-use graphical user interface and bindings with ROS middleware.

## Social robots

The field of social robotics puts focus on the social impacts of robots (as opposed to, for example, how to build a functioning robotic arm). Huang and Moore is an exploratory study on how a social robot’s affordances (its appearance, voice, and behaviors) affect user perceptions in conversational HRI. The researchers found that initial warmth is significantly influenced by static affordances, with child-like robots starting strongly but declining post-interaction. The authors highlight the necessity of aligning affordances (both static and dynamic) with the robot’s intended role and ensuring genuine, responsive interaction to manage user expectations and bridge the “habitability gap” which refers to the discrepancy between a robot’s capabilities and what a user might expect the robot should be able to do.

In a similar line of research, Robb et al. considered how robot morphology affects conversational interaction. The findings indicate that the robot, with its anthropomorphic features and social cues, generally fostered higher engagement and was trusted more in the high-stakes emergency context (in their experiment, an off-shore energy platform with a time constraint to resolve the issue) compared to the voice-only smart speaker. Their work shows that embodiment could be a key factor in successfully deploying conversational agents in the professional workplace.

In Figueroa et al., the authors highlight the growing social issue of loneliness and the burden on healthcare systems, suggesting that social robots could be a promising solution. Key findings from interviews with the participants from the memory clinic and interaction logs indicate that participants gradually accepted the robot, developed a sense of attachment and companionship, and maintained regular usage over several months, suggesting a positive influence on their daily lives.

Continuing on the theme of social robotics, Lumer and Buschmeier explore how expectations of robot politeness can affect interactions between humans and robots. The authors identified two types of politeness: adaptive (i.e., politeness oriented toward an individual listener) and rule-governed (i.e., politeness that follows cultural and societal norms). Their findings indicate that while humans use both types of politeness, users primarily expect robots to exhibit only the functional, rule-governed politeness due to the perceived lack of “feelings” or agency in the artificial agents. This distinction offers valuable design implications for enhancing the user experience of social robots, suggesting that rule-governed politeness is suitable for public settings, while adaptive, customized politeness may be desired in private settings.

When robots interact with humans using spoken dialogue, humans bring expectations that robots have a degree of emotional awareness in the interactions. Mishra et al. investigates the use of Large Language Models (LLMs) for real-time emotion generation in human-robot dialogue. This work highlights the potential of LLMs to move beyond just generating speech by controlling the affective-emotional behavior of robots in real-time, for applications in areas like companionship and customer service.

## Human-robot communication strategies

Recent research is leading to human-robot communication becoming more natural, but there are some important considerations that need to be first addressed. Groß et al. details an empirical study investigating the effectiveness of using negation as a contrastive guidance strategy within explanatory human-robot dialogue for task performance. The study concludes that incorporating negation can enhance the naturalness and effectiveness of robot-to-human explanations, supporting the goal of developing more adaptive and communicative artificial intelligence.

In a similar line of research, Kawakubo et al. explored asymmetries in human-robot communication. The authors define asymmetry as the situation where a robot treats a human like a person, but the human treats the robot like a machine (perhaps surprisingly due to the highly anthropomorphic robots used by the authors). Experiments using simulated dialogue videos indicated that this “pretending to tailor” strategy, while non-human-like, could positively enhance the perception of the robot’s effort to adapt, particularly among customers who already view the robot as a system, thereby proving effective for a human-robot symbiotic society.

Siskind et al. begins with a quote from Arthur C. Clarke: “Any sufficiently advanced technology is indistinguishable from magic” upon which the authors elaborate: “A magician’s trick and a chatbot conversation have something in common: most of their audiences do not know how they work.” The researchers identify several key psychological techniques from magic, such as misdirection, controlling expectations, and emotional connection, and adapt them to a social robot named Haru. The study advocates for leveraging the showmanship and psychological mastery of magicians to create more impressive, satisfying, and memorable interactions with conversational AI.

Li and Ross present a controlled HRI study aimed at systematically invoking and identifying different states of user confusion during task-oriented dialogues. The authors used a Wizard-of-Oz design with a Pepper robot to trigger non-confusion, productive confusion (i.e., when a human user recognizes and attempts to directly resolve the confusion), and unproductive (i.e., when a confusion persists despite attempts at addressing it) confusion states. The work establishes strong correlations between confusion levels and observable features, laying the groundwork for developing more sophisticated, affect-aware strategies for task-oriented HRI systems.

Taken together, robots built on a framework that enables reproducibility, affordances that are useful to humans and embodied morphologies, appropriate politeness and emotional qualities make for more socially acceptable robots. Socially acceptable robots are critical in scenarios that are becoming more commonplace, such as loneliness in aging populations. Moreover, robots that can handle negation, manage asymmetries, be engaging despite limitations, and handle user confusion are all requirements of robots that communicate effectively with humans using dialogue. We hope that readers appreciate the articles in this Research Topic and can build on this line of work.

